# Allogeneic Dentin Graft: A Review on Its Osteoinductivity and Antigenicity

**DOI:** 10.3390/ma14071713

**Published:** 2021-03-31

**Authors:** In-Woong Um, Jeong-Keun Lee, Jun-Young Kim, Yu-Mi Kim, Neema Bakhshalian, Yeong Kon Jeong, Jeong-Kui Ku

**Affiliations:** 1 R&D Institute, Korea Tooth Bank, Seoul 03189, Korea; h-bmp@hanmail.net (I.-W.U.); yumi0825@hanmail.net (Y.-M.K.); 2 Department of Oral and Maxillofacial Surgery, Institute of Oral Health Science, Ajou University School of Medicine, Suwon 16499, Korea; arcady@ajou.ac.kr; 3 Oral Science Research Center, Department of Oral & Maxillofacial Surgery, Yonsei University College of Dentistry, Seoul 3722, Korea; jyomfs@yuhs.ac; 4 Biomedical Implants and Tissue Engineering (BITE), Herman Ostrow School of Dentistry, University of Southern California, Los Angeles, CA 90089, USA; bakhshal@usc.edu; 5 Advanced Periodontology, Herman Ostrow School of Dentistry, University of Southern California, Los Angeles, CA 90089, USA; 6 Department of Oral and Maxillofacial Surgery, Section of Dentistry, Armed Forces Capital Hospital, Armed Forces Medical Command, Seongnam 13574, Korea; ykjung5111@hanmail.net

**Keywords:** allogeneic, antigenicity, bone substitutes, demineralized dentin matrix, osteoinductivity

## Abstract

Studies on allogeneic demineralized dentin matrix (Allo-DDM) implantation in the 1960s and 1970s provided the most reliable preclinical evidence of bone formation and antigenicity in an extraosseous site. Recently, applications of Allo-DDM at skeletal sites were studied, and have provided reliable evidence of bone-forming capacity and negligible antigenicity. However, the osteoinductivity and antigenicity properties of Allo-DDM in extraskeletal sites have not yet been investigated due to the lack of follow-up studies after the initial research. The clinical applications of autogenous DDM (Auto-DDM) have been standardized in some countries. Long-term clinical studies have reported the development of several shapes of Auto-DDM, such as powders, blocks, moldable forms, and composites, with recombinant human bone morphogenetic protein-2. For the development of Allo-DDM as a reliable bone graft substitute next to Auto-DDM, we reviewed preclinical studies on the bone induction capacity of allogeneic dentin at extraskeletal as well as skeletal sites. Electronic databases were screened for this review in January 2020 and searched from 1960 to 2019. This review aims to provide a foundation on the preclinical studies of Allo-DDM, which could enable future researches on its osteogenic capability and antigenicity. In conclusion, Allo-DDM showed great potential for osteoinductivity in extraskeletal sites with low antigenicity, which neither adversely affected osteogenic capability nor provoked immunologic reactions. However, the risk of viral disease transmission should be researched before the clinical application of Allo-DDM.

## 1. Introduction

Dentin is a cell-free matrix without vascularization, while bone includes osteocytes and vessels. The organic and inorganic components of dentin and bone consist of similar components, such as biological apatite (HAp: 70%), collagen (18%), non-collagenous proteins (NCPs, 2%), and body fluid (10%) in weight by volume [1]. Dentin was reported to have a bone-inducing function in a study from 1967, and demineralized dentin matrix (DDM) was revealed to be an osteoinductive and osteoconductive collagen material with less antigenicity; it enabled the release of growth factors such as bone morphogenic proteins (BMPs) [2,3].

The general method for producing DDM, which is an acid-insoluble type I collagen showing a high degree of cross-linking with collagenous and matrix-binding proteins such as transforming growth factors (TGFs), insulin growth factor, fibroblast growth factor (FGF), and bone morphogenetic proteins (BMPs), involves crushing dentine, after removing the cementum and enamel, into a powder and demineralizing it [1,4,5,6].

Demineralization is a necessary process for DDM to act as a bone substitute because HAp inhibits the release of growth factors, and carrying out this process does not lead to the degradation of these growth factors [1].

Major osteoinductive growth factors, similar to dentin-matrix-derived BMPs, have been identified in rats [7], bovines [8], rabbits [9], and humans [10]. The molecular weight of human dentin-matrix-derived BMP was estimated to be approximately 20.0 kDa by SDS-PAGE and its pH was found to be 8.8 by isoelectric focusing; these values somewhat resemble those of bone-matrix-derived BMPs [10].

Accordingly, several forms of autogenous DDM (Auto-DDM) (e.g., powder and blocks) have been developed and their clinical safety and effectiveness in implant dentistry have been tested [4]. An Auto-DDM powder was first applied for maxillary sinus augmentation in humans in 2003 [11]. In 2006, Gomes et al. [12] conducted the first clinical study involving humans with Auto-DDM slices at a thickness of 8 µm. They reported that bone formation was higher with Auto-DDM than with the negative control (empty) and polytetrafluoroethylene membrane. Since then, studies on the regenerative potential of Auto-DDM blocks, including clinical studies involving humans, for guided bone regeneration (GBR), socket preservation (SP), and sinus augmentation have been reported [13,14,15,16,17,18].

The applications of Auto-DDM, as an alternative to autogenous bone grafts, have shown promising clinical and histological results for SP and GBR in implant dentistry owing to its inherent osteoinductive and osteoconductive capacity [13,14,15,16,17]. However, Auto-DDM has limitations despite its proven bone-formation capacity: (1) dependence of the Auto-DDM quantity on the number of teeth indicated for extraction and the condition of the extracted teeth, (2) lack of a standard method to process Auto-DDM, and (3) patient preference. Therefore, the application of dentin graft material from other individuals—allogeneic DDM (Allo-DDM)—has been considered as an alternative to Auto-DDM [19,20].

Allo-DDM was conceptualized from the demineralized bone matrix (DBM), which was largely developed and defined for the bone induction principle (BIP), which states that a protein macromolecule in dentin and bone induces the differentiation of mesenchymal cells into osteoblasts; this was postulated by Urist in 1965 [2,20]. The DBM is a refined allograft that has osteoinductivity and has been clinically used since the 1980s. However, many studies have indicated that the osteoinductive properties of DBM can be affected by several factors, such as donor age, gender, particle size, and methods of preparation, sterilization, and storage [21,22].

Although Auto-DDM is commonly applied in some countries such as Korea, India, and Japan, very few studies have investigated the application of Allo-DDM as bone substitutes for bone graft surgery with regard to its osteoinductivity and antigenicity. This narrative review aims to summarize the preclinical evidence on the osteoinductivity and antigenicity of Allo-DDM and to provide future directions for research on the clinical safety and efficacy of Allo-DDM in maxillofacial bone regeneration.

## 2. Methods

**Figure 1:**
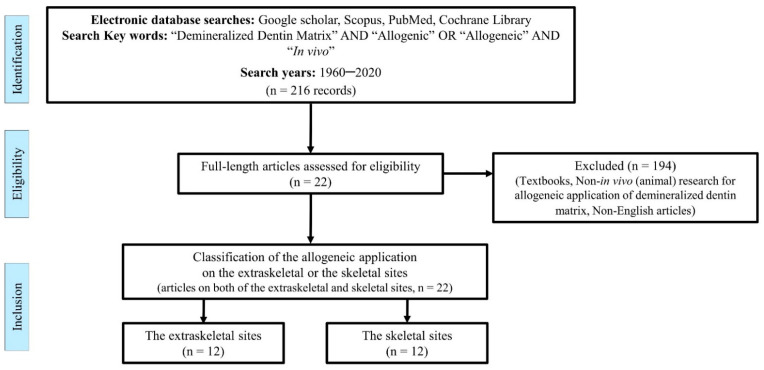
Flow diagram for the review process.

Google Scholar, Scopus, PubMed, and the Cochrane Library databases were screened for this review in January 2020. The years searched were from 1960 to 2019, using the keywords “Demineralized Dentin Matrix” AND (“Allogenic” OR “Allogeneic”) AND (“In vivo” OR “Animal”). The relevant full-length articles were obtained from the electronic databases, and the authors read and selected relevant studies for review according to the following inclusion criteria: (1) articles written in English in a peer-reviewed journal, (2) any in vivo (animal) studies that included any of the search keywords, and (3) articles that focused on osteoinduction or antigenicity. The exclusion criteria were: (i) articles for which full-text was not available, (ii) articles that did not pertain to the allogeneic application of demineralized dentin matrix, and (iii) classifications described in a textbook. The final articles selected were classified based on the application of the extraskeletal and the skeletal sites after the inclusion and exclusion criteria were applied (Figure 1).

## 3. Results

**Figure 2:**
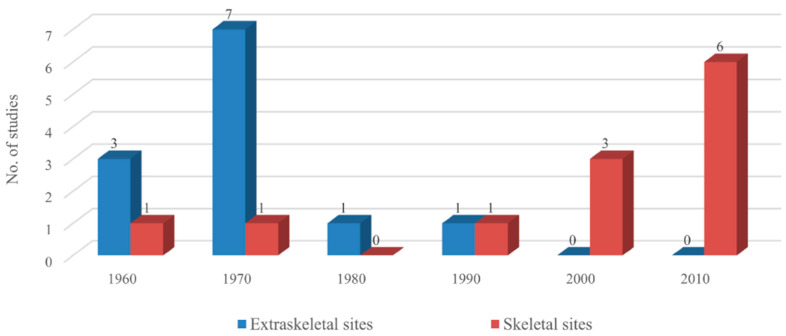
Total number of animal studies on allogeneic dentin grafts across decades. Preclinical in vivo studies on the implantation of allogeneic dentin grafts at the extraskeletal sites began in the 1960s along with the development of bone induction principle (BIP) [21,22], peaked in the 1970s, and then decreased until the 1990s. Since the 2000s, the number of in vivo studies on allogeneic dentin grafts at skeletal sites has begun to increase abruptly.

Among the selected articles, twelve in vivo studies evaluated the osteoinductivity of allogeneic dentin at extraskeletal sites of the abdominis muscle or subcutaneous pockets in rabbits, guinea pigs, and rats mainly during the 1960s and 1970s (Table 1) [23,24,25,26,27,28,29,30,31,32,33,34]. Twelve other in vivo studies evaluated the bone-forming capacity of Allo-DDM in skeletal defects after the 1990s (four studies were conducted using alveolar defects in rats, rabbits, and monkeys [24,34,35,36]; seven used calvarial defects in rabbits and mice [37,38,39,40,41,42,43]; one used femoral defects in rabbits [44]). Two of these twelve studies were performed both in the muscles and alveolar bone (Table 2) [24,34]. As the preclinical in vivo studies on the implantation of allogeneic dentin grafts at the extraskeletal site began in the 1960s along with the development of BIP, most Allo-DDM research had stopped in the 1980s, when the clinical application of DBM began. Since the 2000s, the number of in vivo studies on allogeneic dentin grafts at skeletal sites for alveolar bone repair in implant dentistry began to increase (Figure 2).

## 4. Discussion

### 4.1. Osteoinductivity

Most of the studies evaluated the osteoinduction property of dentin with regard to histological, radiological, and biochemical outcomes at the extraskeletal sites (Table 1).

**Figure 3:**
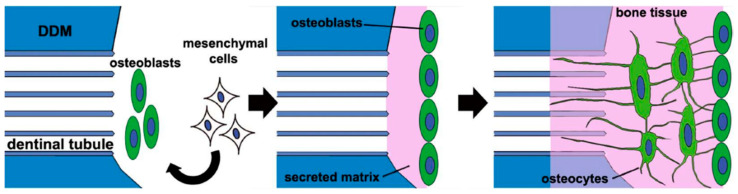
A schematic of the new bone formation process after human demineralized dentin matrix (DDM) transplantation in rat skeletal defects [19]. When the human DDM is transplanted into the rat calvarial defect, a small amount of bone morphogenic proteins (BMPs) that are gradually released from the DDM induce mesenchymal cells to differentiate into osteoblasts. The osteoblasts secrete the matrix and form a new osteoid with embedded osteocytes, which are buried osteoblasts. The osteocytes then form a network on the DDM surface, with some of them extending into the dentinal tubules.

In 1967, Bang and Urist [23] first reported bone induction at 4 weeks after Allo-DDM implantation without causing inflammation or foreign body reactions in the abdominal muscle of rabbits and rats. After 12 weeks, the new bone was remodeled into the bone marrow without a solid bone matrix [24]. Since then, many researchers have revealed that Allo-DDM induced bone formation in extraskeletal sites of rats and rabbits, and produced a high yield of new bone and cartilage in volumes that seemed to be proportional to that of the original grafts [23,24,25,26,27,28,29,30,31,32,33,34,35,40,41,43]. This inductive substrate, which is similar to DBM owing to its ability to allow the differentiation of fibroblasts from mesenchymal cells into cartilage or bone [2,25], is derived from the extracellular components of the dentin matrix and not from cytoplasmic proteins, which are dispersed in the ground substrate or extracellular material among the inducing and responding cells of the recipient (Figure 3) [19].

The qualitative trends of cellular sequences after the implantation of Allo-DDM could be as follows: (1) inflammation, (2) vascularized connective tissue formation, (3) erosion, (4) recalcification, and (5) bone formation [31]. Nilson [32] summarized the cellular events during induced bone formation as follows: (i) resorptive reactions mediated by monocytes, macrophages, and dentinoclasts, (ii) fibroblastic reaction, as an unspecific encapsulation process, and (iii) osteoblastic reaction with osteoid formation (Table 1).

The sequences of cellular transformation following the implantation of Allo-DDM suggest that the graft is invaded by the vascular “mesenchyme” with a brief inflammatory reaction. Some of the mesenchymal cells became multinucleated giant cells that proceed to erode tunnels in the matrix and enlarge the pre-existing cavities (the dentinal tubules). The matrix around the eroded chambers is then re-calcified, presumably due to the diffusion of mineral ions from the new blood vessels. Osteoblasts then replace the multinucleated cells on the eroded and calcified surfaces, which start to deposit the bone matrix and cement line [27,45]. In extraskeletal sites, Allo-DDM showed penetration into the bone and was resorbed slower than DBM, presumably because DDM is a denser collagen matrix, and has neither vascular channels nor marrow space [26]. The new bone induced by Allo-DDM was almost twice the size of the decalcified cortical bone graft [24,29].

In general, osteoinduction is a surface-oriented reaction that does not involve the deep, relatively non-available structures of the matrix [2,46]. In an extraction socket as a four-wall skeletal defect, the induced bone produced a separate unattached ossicle inside the bone cavity. The process of bone regeneration from the pre-existing cavities and bone induction from DDM were separated and delineated by a fibrous envelope. On the other hand, in the mandibular critical-sized defect, the induced bone from DDM was not separate from the recipient bone and showed a generally interwoven and continuous pattern [24]. Regardless of whether the induced bone from the graft and host was separate or continuous in the skeletal defect, a separate unattached ossicle was produced inside the bone cavity. After 12 weeks, a large part of the structure of the final tissue profile was that of a cancellous bone and not a solid bone tissue [24,35].

Gomes and his colleagues [36,37,38,42] reported that new bone formation on implantation with Allo-DDM in rabbit skeletal defects was greater than that in ungrafted defects. Um et al. [43] reported that bone induction by Allo-DDM was interwoven and continuous with the recipient bone. In 2018, Tanoue et al. [19] suggested that in the new bone formation process, after xenogeneic transplantation with human DDM in rat calvarial bone defects, a small number of BMPs were gradually released from the DDM, induced mesenchymal cells to differentiate into osteoblasts which secrete, and formed a new osteoid on the DDM surface (Figure 3).

**Figure 4:**
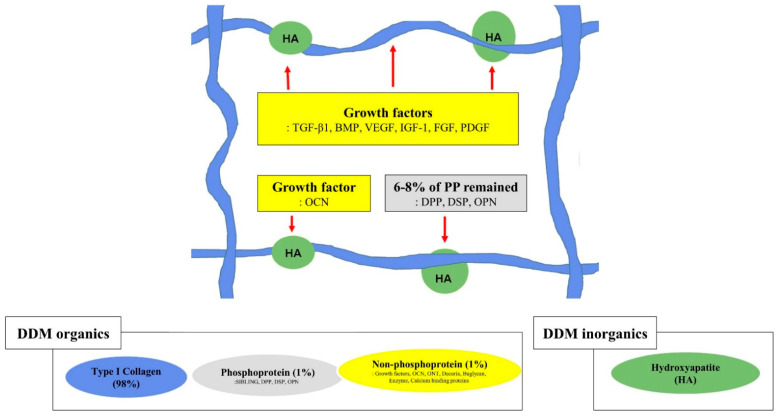
Graphical illustration of the structural relationships among the components of the extracellular matrix on demineralized dentin matrix [1,7,48]. Collagen and acid-insoluble non-collagenous protein networking. Type I collagen (in blue), hydroxyapatite (in green), non-phosphoprotein (in yellow), and phosphoprotein (in gray). The red arrow indicates hydroxyapatite binding; the red dotted arrow indicates collagen binding. SIBLING—small integrin-binding ligand, N-linked glycoprotein; DPP—dentin phosphoprotein; DSP—dentin sialoprotein; OPN—osteopontin; VEGF—vascular endothelial growth factor; BMP—bone morphogenetic protein; OCN—osteocalcin; IGF-1—insulin-like growth factor 1; FGF—fibroblast growth factor; PDGF—platelet-derived growth factor.

Bone morphogenetic activity in the DDM indicates that BMPs reside in or on the quaternary structures of collagen fibrils, or the protein core of proteoglycans [1,43]. During the demineralization process, by using ethylenediaminetetraacetic acid (EDTA), hydrochloric acid (HCl), and lactic acid, BMPs were found to be more stable in the dentin than the bone because of the highly cross-linked structure of the fibrous (insoluble) protein and high density of collagen in the dentin matrix [25,26,47]. However, BMPs are heat-stable and resistant to strong acid but can be destroyed by ultraviolet (UV) irradiation and dilute solutions of sodium hydroxide (NaOH) [2,22,25,26]. However, DDMs retain the BMP activity in the insoluble organic matrix (98% collagen) after the removal of most of the soluble components, as the collagen fibril may be the locus of BMPs (Figure 4) [1,7,8,9,10,48]. Recently, enzyme-linked immunosorbent assay quantification of growth factors in human dentin indicated the predominance of TGF-β1 (15.6 ng/mg of DDM), with relatively lower concentrations of BMP-2 (6.2 ng/mg of DDM), FGF (5.5 ng/mg of DDM), vascular endothelial growth factor (5.0 ng/mg of DDM), and platelet-derived growth factor (4.7 ng/mg of DDM) [1]. Consequently, Allo-DDM showed great bone morphogenetic activity with growth factors as osteoinductive property in extraskeletal sites and bone healing capacity in skeletal sites.

### 4.2. Antigenicity

A few studies assessed the levels of antigenicity by immunologic reaction markers such as histocompatibility, second-set reaction of skin grafts in extraskeletal sites, and white blood cell (WBC) count in skeletal sites (Table 1 and Table 2).

At the extraskeletal site, histocompatibility antigens in Allo-DDM were first investigated in 1968 [25]. Weaker antigens produced only a thin wall of inflammatory tissue and caused only a brief delay in the onset of inductive interaction of mesenchymal cells. The tolerance and biological activity could be enhanced by preliminary treatment of Allo-DDM with the combination of lyophilization and co-radiation, which inactivates the histocompatibility antigens in the allogeneic dentin matrix. The inductive activity of the treated matrix could be retained by using chloroform and methanol to remove nearly all lipoproteins and lipids (Table 1).

In an experimental model with the rejection reaction in skin allografts in 1972, Bang [28] reported that Allo-DDM might have some tissue antigens that could evoke an immune response in the host, resulting in a decreased survival time of the skin allografts [28,34,40].

When used in skeletal defects, Allo-DDM showed no or low antigenicity at the tissue level [34,36,37]. Except for the initial inflammatory reaction, no immunological rejection response or foreign body reaction was observed with the Allo-DDM graft [41]. The mean WBC count was higher in the Allo-DDM group than in the negative control group at two days postoperatively but reached equivalence at postoperative days 15 through 90 (Table 2) [41]. Even the different WBC results of both the groups were in the range of that of a homogeneous group without immunologic symptoms [49]. Therefore, this initial inflammatory reaction of Allo-DDM could not be associated with an immunologic reaction and did not inhibit the osteoinductivity of Allo-DDM [2,30].

Some BIPs are lost in DBM with lyophilization, irradiated, or heating processes [2]. Several methods, including sequential chemodigestion and chemosterilization, for antigen depletion, have been utilized to reduce the host immune response while preserving the osteoinductive properties [2,50]. Allogeneic reactive glycopeptides in the DBM, derived from osteocytes or other cell membranes in the marrow component, can elicit an immune response through indirect antigen presentation [51]. A vital dentin might have allogeneic immune components, such as cytoplasmic membrane antigens, odontoblastic dentin processes, and cementocyte membranes of cementum [52]. In conclusion, owing to the acellular and avascular nature of the dentin matrix, which does not induce antigenicity [3], DDMs have low antigenicity [3,22], but this insignificant antigenic effect from the potential immune components could possibly lead to reduced osteogenesis [23,28,34,40].

### 4.3. Demineralization of Dentin Matrix

In many studies, complete demineralization of the dentin matrix, until a calcium-free state, is achieved using 0.25–0.5 M EDTA and 0.2–0.6 N HCl [23,24,25,26,27,28,29,30,31,32,33,34]. Among the several demineralization protocols, the treatment with 0.6 N HCl led to the most effective osteoinductivity, as revealed by histological and roentgenographic examinations in rats and rabbits after 4–12 weeks of implantation [25]. The osteoinductive capacity was not different between demineralization with 0.2 and 0.6 N HCl. Although chelating agents such as EDTA have deleterious effects on bone [25], Bang [30] argued that no definite difference in osteoinductivity existed between dentin demineralized with HCl and EDTA. According to Glowacki [22] and Russell et al. [53], demineralization with 0.1 N EDTA had detrimental effects on the osteoinductivity of bone implants.

The minerals from the dentin matrix insulate the BMPs and interfere with the transmission of the bone morphogenetic property [1,24,53]. Demineralization of the dentin matrix not only contributes to removing allogeneic immune components including minerals and acid-soluble proteins but also to opening the dentinal tubules [29,45]. Additionally, after demineralization, the widened nanoporous dentinal tubules and exposed collagen fibers could help in the release of the dentin-matrix-derived growth factors, resulting in the proliferation of mesenchymal cells, activation of collagenolytic enzymes, the transformation of fibroblasts to osteoblasts, favorable cell attachment, and osteoinduction [1,5,6,24,53,54,55].

With regard to partial demineralization, a study from 1998 on human DDM as a carrier for recombinant human BMP-2 reported that partially demineralized dentin matrix (partial-DDM, % not specified) did not cause osteoinduction on allogeneic transplantation into the muscle of mice [56]. On the other hand, in 2018, a similar study of partial-DDM in rabbits showed bone induction in both the subcutaneous tissue of mice and the skeletal defect of rabbits [43]. Koga et al. [57] showed superior bone regeneration with partial-DDM (70% demineralization) than that with complete-DDM (complete-DDM) in rat skeletal defects. Partial-DDM can contain more growth factors that promote osteogenesis than complete-DDM since many NCPs are released from the dentin matrix during complete demineralization [57]. Controversies still exist regarding the ideal demineralization degree of DDM owing to the scarcity of related research; however, such information is available for extrapolation from the research on DBM [53]. The demineralization agents and the time used to make the DDM affect the mineral percentage of the resulting DDM. The DDM in powder form has a mineral content of about 5–10%/volume, while DDM in block form has a mineral content of about 10–30%/volume with approximately 90%/volume of type I collagen [4,43,58] (Figure 4).

Many researchers found that undemineralized allogeneic dentin matrix (Allo-MDM) did not induce alkaline phosphate activity and cartilage or bone formation in the extraskeletal sites [27,28,29,31,32]. Allo-MDM required a lag phase of 8 to 12 weeks to produce a scanty deposit of bone in 75% of the grafted area [23,24,30]. The resorption of Allo-MDM was always incomplete and delayed, whereas osteogenesis was induced at 4 weeks after the implantation of Allo-DDM.

However, in rabbit skeletal defects, Allo-MDM acted as a three-dimensional osteoconductive scaffold contrary to the results obtained in the extraskeletal sites [24,27,30,39,44]. Histomorphometrically, the bone regeneration capacity of Allo-MDM was similar to that of autogenous bone grafts [44]. In mice with skeletal defects, Allo-MDM slices were found to have been completely vascularized at 22 days postoperatively and osseointegrated within 12 weeks, similar to autogenous bone, ß-tricalcium phosphate (ß-TCP) scaffolds, and ungrafted sites [39]. Nonetheless, in a recent in vivo study, human DDM showed superior bone healing than MDM in the skeletal defects of rats [59].

Therefore, MDM appears to act as an osteoconductive scaffold; however, it has poor bone formation capacity or is rejected in extraskeletal sites, which requires the activation of inducible osteogenic precursor cells (IOPCs). According to Friedenstein et al. [60] and Owen [61], at the extraskeletal tissue, osteogenesis occurred only in the presence of IOPCs, which need an inducer from the demineralization or osteoclastic resorption of the dentin matrix.

In summary, the demineralization with 0.2–0.6 N HCl showed the most effective osteoinductivity of Allo-DDM. With regard to the degree of demineralization, partial-DDM was superior for bone-forming outcomes in comparison with complete-DDM, since many endogenous growth factors could be lost during complete demineralization.

### 4.4. Geometry of Allo-DDM

The osteoinductivity of Allo-DDM at extraskeletal sites was not affected by its various shapes and sizes, including pieces of 2 × 2 × 1 mm^3^ [28,30,32], whole root dentin blocks [29,31], and dentin rolls [33]. Other geometric structures of Allo-DDM include whole dentin or 1.0 mL or 3 mm^3^ of dentin used by Urist and colleagues [23,24,25,26], and coarse powders of 200–300 µm and granules of 1 mm^3^ introduced by Reddi et al. [27] and Pinholt et al. [34] (Table 1). Reddi et al. [29] conducted an experimental study that implanted teeth in the rat subcutaneous tissue and showed the transformation of fibroblasts into the cartilage and bone tissues at the end of the tooth root, where it allowed the capillary penetration from the subcutaneous tissue. However, cartilage formation was observed inside of the root, probably because of the lower oxygen tension in this zone. When capillaries were provided access to both the ends of the root by cutting the other end, bone was formed at both ends with cartilage in the middle. In mineralized tooth implantation, a cavity inside the tooth was populated with fibroblasts that failed to differentiate into bone and cartilage [29,62].

**Figure 5:**
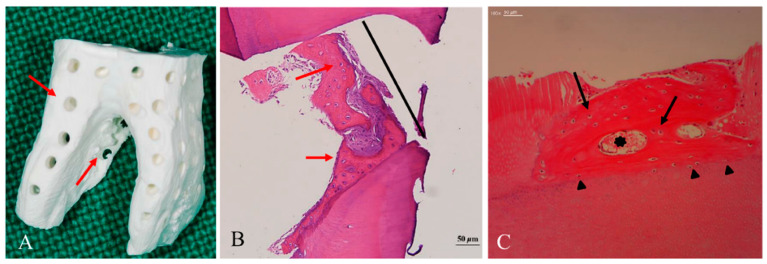
Histological findings of dentin block grafts into the skeletal sites [63]. (**A**) Macropores (300 µm, red arrow) on the dentin block that penetrated from the surface to the pulp space provided the space for vascular invasion. (**B**) At 8 months after the graft, the macropores (300 µm, black line) were filled with newly formed osteoids with embedded active chondrocyte-like cells (red arrow) that closely contacted the inner wall of the macropore. (**C**) At 3 months after the graft, a newly formed osteoid, which had osteocytes (black arrow) and vessels (black asterisk), had been deposited on the dentin block surface. Cellular fusion without fibrous tissue invasion was observed on the border between the osteoid and the dentin matrix (black arrowhead).

In skeletal sites, Allo-DDM showed bone formation capacity regardless of shape and size (Table 2) [24,34,35,36,37,38,39,40,41,42,43,44]. Macroporous (200–300 μm) human DDM blocks, that completely penetrated the whole DDM, provided the space for vascular invasion, resulting in osteoconductive bone formation and osteoinductive deposits of new osteoids on the DDM surface (Figure 5) [63]. A 500-µm macroporous human DDM block was more effective for bone formation than non-perforated DDM in the rabbit skeletal sites [64]. A 1000-µm macroporous human DDM block showed new bone formation on the entire DDM in the skeletal defects of sheep [65]. These results indicated that the geometric structure of human DDM could contribute to active bone ingrowth in critical-size bone defects [65].

With regard to the particle size of DDM, the only studies available are regarding DDM powders (particle size, 200–400 µm) in 1970 and 1 × 1 × 1 mm^3^ granules in 1990 [29,34]. Most subsequent studies used a DBM size that might have a similar influence on the transformation of fibroblasts into osteoblasts [2,29,62,66]. DBM powder with a particle size of 420–850 µm showed the maximum effect on local fibroblasts for the induction of endochondral bone, whereas DBM with smaller particles (≤74 µm) delayed cartilage formation with scanty chondroblasts [62]. Another study compared three different DBM particle sizes, and concluded that large particle sizes of 500–1000 µm were desirable when the DBM was implanted alone, whereas small particles (<500 µm) were recommended in conjunction with mesenchymal stem cells [67]. In the 2010s, Allo-DDM powders with a particle size of 300–800 µm showed excellent bone-forming capability in skeletal defects [40,43]. Recently, Koga et al. [57] reported that human DDM (70% demineralized) with a large particle size (1000 μm) showed superior bone regeneration than that with small particle sizes (180–212 and 425–600 μm), which was consistent with the findings of previous studies [34,44]. Nam et al. [68] compared two different sizes of human DDM particles (250–1000 vs. 1000–2000 µm) and concluded that smaller particles were more effective in promoting osteogenesis.

Dentinal tubules in dentin (20,000–60,000/mm^3^, approximately 3 µm diameter) are a unique spatial nanoporous structure that can be enlarged to microporous geometric structures by the demineralization process, resulting in increased porosity from 3% to 20% [25,54,55,69]. This modified geometry of DDM can facilitate the release of the dentin-matrix-derived growth factors, such as BMPs inside the dentin matrix, and hydroxyapatite-binding proteins, as well as the influx of proteins from host tissues [45,54,70].

In 2018, Tanoue et al. [19] reported that the transplantation of human DDM into rat skeletal defects caused the osteocytes embedded in the newly formed bone to create a network on the DDM surface with a connection into the enlarged dentinal tubules (Figure 3). This finding was consistent with those of fundamental studies conducted in the 1960s that showed macromolecular networks between the dentinal tubules and newly deposited osteoids [24,25].

## 5. Conclusions

This article represents the first comprehensive review focused on the osteoinductivity and antigenicity of Allo-DDM. Allo-DDM has demonstrated a great potential for osteoinductivity in extraskeletal sites. Allo-DDM showed low antigenicity, which neither adversely affected osteoinductivity nor provoked immunologic reactions. Owing to the limited amount of research related to Allo-DDM and the lack of follow-up studies after the initial research, there has been no clear evidence to support the free antigenicity of Allo-DDM due to the potential immune components from vital dentin, which might be removed during demineralization process. Further, even though acellular and avascular dentin matrix cannot be a carrier for a virus, safety from the risk of viral disease transmission has not been mentioned so far in the condition of in vivo. Future studies should investigate the optimization of the processing methods and the geometry of Allo-DDM, which plays an important role in its osteoinductivity and osteoconductivity. Furthermore, the risk of viral disease transmission should be researched in vivo before the clinical application of Allo-DDM.

## Figures and Tables

**Table 1:** Summary of preclinical evidence on bone induction capacity of allogeneic dentin at extraskeletal sites.

Year (Author) [Ref.]	Theme	Donor (Tooth)	Geometry (Dentin)	Processing Method	Implant Site/Time	Key Findings
1967 (Bang & Urist) [23]	Osteoinduction	Rabbit (Mature molar)	Whole root dentin	Complete demineralization (0.6 N HCl)	Abdominis muscle/12 weeks	Demineralized dentin (DDM): Similar to the matrix of bone, induces bone formation at 4 weeks proportional to the volume of the original implant.
		Rat (Mature molar)		Un-demineralization	Abdominis muscle/12 weeks	Undemineralized dentin (MDM): Does not begin to be resorbed until 8–12 weeks later than demineralized dentine.
1967 (Yeomans & Urist) [24]	Osteoinduction	Young New Zealand rabbit (Dentin)	Grinding dentin 1.0 mL	Complete demineralization (0.6 N HCl)	Rectus abdominis muscle/4, 8, 12 weeks	DDM: Rapidly resorbed more than bone, induces bone in 100% of implants within 4 weeks (^3^H-glycine autoradiograph: Positive evidence of translocation of ^3^H-glycine from the donor tissue to the cells of the host bed. Figure 2 in text)
				Un-demineralization	Rectus abdominis muscle/4, 8, 12 weeks	Undemineralized dentin (MDM) induces bone in 75% of implants, but only after a latent period of 8–12 weeks.
1968 (Urist et al.) [25]	Inductive substrate antigenicity (HCl vs. EDTA)	Rabbit (Dentin)	Grinding dentin 1.0 mL	Demineralization (0.6 N HCl)	Abdominis muscle/4–12 weeks	Inductive substrate such that new bone formation is the best at 0.6 N HCl and 150 mmol NaCl, and is derived from the extracellular and not cytoplasmic proteins. Antigenicity: The immune response is lowered, and the inductive activity appears earlier with lyophilized and irradiated ^60^Co at the dose of 0.2 Mrads or less.
		Rat (Dentin)		Demineralization (0.25 M EDTA)		EDTA: The inductive activity had been eliminated only from the exposed surface. Bone: The inductive activity was completely destroyed, Dentin: Persisted inductive activity for a longer time.
1970 (Huggins & Urist) [26]	ALP activity	Rat (Incisor)	Whole root dentin	Complete demineralization (0.5 N HCl, 1 mL/mg)	Rat abdominis muscle/1 year	Alkaline phosphatase activity (ALP) within 24 h: Reached maximum on day 7. Matrix transformation: Cartilage appeared within 5 days; bone and bone marrow formed within 14 days. The induced cartilage disappeared within 5 weeks. Transforming effects in postnatal life are comparable to embryonic induction because of highly cross-linked, exceptional resistance to the deleterious action of strong acid of dentin matrix.
1970 (Huggins et al.) [27]	Transformation of fibroblasts	Rat (Incisor) Mice (Incisor)	Dentin powder (pooling) 200–400 µm (10–15 mg)	Complete demineralization (0.5 N HCl)	Subcutaneous pockets/9–21 days	DDM: Active transformation. An early and intense attraction for fibroblasts of the subcutaneous tissue.
				Un-demineralization		MDM: Does not induce ALP, cartilage, or bone.
		Guinea pig (Incisor)	Dentin powder (Pooling) 200–400 µm (10–15 mg)	Complete demineralization (0.5 N HCl)	Subcutaneous pockets/9–21 days	DDM: No bone induction in guinea pig Transformation of fibroblast: Rat, mouse > guinea pig
				Un-demineralization		MDM: Does not induce alkaline phosphate, cartilage, or bone.
1972 (Bang) [28]	Antigenicity	Guinea pig (Incisor)	Root dentin pieces 2 × 2 × 1 mm	Demineralization (0.2 N HCl)	Abdominis muscle/4–12 weeks	Antigenicity: Histological examination 1. Demineralized and undemineralized dentin could evoke an immune reaction. 2. The first set of Allo-DDM induces bone formation in high percentages of cases. 3. Induction was reduced in 2nd set of grafts 4. No difference was observed in bone-inducing capacity between DDM and lyophilized DDM.
		Guinea pig (Molar)	Pieces 2 × 2 × 1 mm	Un-demineralization		Osteoinduction is prevented in the 2nd set of implants in MDM.
1973 (Reddi & Huggins) [29]	Role of geometry	Rat (Incisor)	Whole tooth root	Demineralization (0.5 N HCl)	Subcutaneous tissue/7–35 days	Cartilage–chondrolysis–osteogenesis–ossicle with hemopoietic marrow. Figure 3a,b in Text
			Whole tooth root	Un-demineralization		Bone and cartilage: Not observed. Finding of fibrosis: Consistent with hypoxic environment of pulp chamber. Figure 3c in Text
1973 (Bang) [30]	Osteoinduction (HCl vs. EDTA)	Guinea pig (Molar)	Molar dentin 1/2 root 6–7 × 3 × 1.5 mm, 30 mm^3^, 30 mg	Demineralization (0.6 N HCl, 0.5 M EDTA)	Abdominis muscle/4–12 weeks	HCl, DDM: 13 out of 30 implants induce bone formation EDTA, DDM: 12 out of 30 implants induce bone formation
				Un-demineralization		MDM: Bone induction process is retarded, and the yield of new bone is low.
		Rat (Molar, Incisor)	Whole molar tooth bud		Abdominis muscle/4–12 weeks	Tooth bud: No induction
			Incisor dentin 6–7 × 3 × 1.5 mm, 30 mm^3^, 30 mg	Demineralization (0.6 N HCl, 0.5 M EDTA)		HCl DDM: 1 out of 12 implants induce bone formation EDTA DDM: 3 out of 12 implants induce bone formation No distinct differences in the bone-inducing capacity of HCl and EDTA demineralization.
			Incisor dentin 6–7 × 3 × 1.5 mm, 30 mm^3^, 30 mg	Un-demineralization		3 out of 24 MDM: Osteoinduction after 12 weeks, bone induction process was retarded, and the yield of new bone was low
1975 (Linden) [31]	Osteoinduction cell sequences	Ash-Wistar rat (Incisor)	Whole root dentin block	Demineralization (0.6 N HCl)	Abdominis muscles/40 days	Cell Sequences in bone induction Qualitative trends may be listed as: (1) inflammation; (2) formation of vascularized connective tissue; (3) erosion; (4) recalcification; and (5) bone formation.
1977 (Nilsen) [32]	Cell reaction	Guinea pig (Molar)	Root dentin Piece 6–7 × 3 × 1.5 mm	Demineralization (0.2 M HCl)	Abdominal muscles/22 days	Induce osteoid formation: Resorption of DDM is a prerequisite for osteoid formation (1) Matrix resorptive reaction mediated by monocytes, macrophages, and dentinoclasts. (2) Fibroblastic reaction as an unspecific capsulation process. (3) Osteoblastic reaction with osteoid matrix formation
1986 (Inoue et al.) [33]	Chondrogenesis in the muscle, skin, periodontal ligament, and bone marrow	Wistar rat (Incisor)	Dentin rolls One incisor-one implant (not pool)	Demineralization (0.6 N HCl)	Abdominal muscles pouch/21 days	Cartilage is formed at 7 days Rectus abdominis muscle (cartilage induce) > Chest subcutaneous tissue (cartilage induce) > Periodontal ligament (deposits of cartilage were not seen)
					Subcutaneous pocket in the chest/21 days	Cartilage is first found at 10 days
					Periodontal ligament of the first molar/21 days	Cartilage was not seen
					Bone marrow in the femur/21 days	Woven bone is found at 10 days
1990 (Pinholt et al.) [34]	Osteoinduction	Male Wistar rat (Incisor)	4 Granules1 × 1 × 1 mm	Demineralization (0.2 N HCl)	Abdominis muscle/4 weeks	Dentin induced new bone formation in 100% of implants No inflammatory or foreign body reactions were observed.

Abbreviations: DDM—demineralized dentin matrix; MDM—mineralized dentin matrix; HCl—hydrochloric acid; EDTA—ethylene diamine tetraacetic acid; ALP—alkaline phosphatase activity; PRP—platelet-rich plasma.

**Table 2:** Summary of preclinical evidence on the bone-forming capacity of allogeneic dentin at the skeletal site.

Year [Author]	Theme	Donor (Tooth)	Geometry (Dentin)	Processing Method	Implant Site/Time	Key Findings
1967 (Yeomans & Urist) [24]	Osteoinduction	New Zealand rabbit	Grinding Dentin 1.0 mL	Complete demineralization (0.6 N HCl)	Mandibular drill hole (ø 5 mm)/4, 8, 12 weeks	DDM: Induce osteogenesis, not dentinogenesis. (1) Bone formed by extension of proliferating osteogenetic cells from the host bed and (2) bone formed by induction inside of dentine matrix are generally interwoven and continuous processes. The old DDM: Resorbed and partially refilled with new bone within 4 weeks, more slowly than bone; produced a separate and unattached ossicle inside the cavity. After 12 weeks, the end product is bone marrow and not solid bone tissue.
					Extraction socket/4, 8, 12 weeks	DDM: Induce osteogenesis and not dentinogenesis. The processes of (1) The regeneration from host bed and (2) the osteoinductive new bone formation from the dentin matrix generally progressed separately. Bone induction is the same as implants in the abdominal wall.
				Un-demineralization	Mandibular drill hole (ø 5 mm)/4, 8, 12 weeks	MDM: New bone formation in 75% of implants only after a latent period of 8–12 weeks. Same results with (23, 24) in soft tissue
					Extraction socket/4, 8, 12 weeks	MDM: New bone formation in 75% of implants only after a latent period of 8–12 weeks. Same results with (23, 24) in soft tissue
1990 (Pinholt et al.) [34]	Osteoinduction	Male Wistar rat (Incisor)	Granules 1 × 1 × 1 mm	Demineralization (0.2 N HCl)	Premaxilla, alveolar ridge (subperiosteal)/ 4 weeks	All 10 DDMs: Induced new bone formation Tissue response: No inflammatory or foreign body reactions were observed
1972 (Bang et al.) [35]	Osteoinduction Osteoconduction	16 Java monkeys	Root dentin pieces 4 × 1 × 1 mm,	Demineralization (0.2 N HCl)	Mandibular defect (ø 7 mm)/1 week–1 year	DDM: Osteoinduction and osteoconduction in histologic study
2004 (Carvalho et al.) [36]	Osteopromotion	36 adult rabbits (Central incisor)	Slices, 8 mm thick (Consisting of enamel, dentin, and cementum)	Complete demineralization (0.6 N HCl)	Mandibular defect (ø 5 mm and 2 mm in depth)/Approx. 13 weeks (90 days)	DDM slices: Biocompatible, stimulated newly formed bone until 30 days after implantation; resorbed during the bone remodeling process. The volume of the newly formed bone is significantly greater in the dentin graft than in ungrafted negative controls with low antigenicity during 13 weeks in a histologic study
2007 (Gomes et al.) [37]	Osteopromotion	48 New Zealand rabbits. Central incisors	Slices, 8 mm in thickness (Consisting of enamel, dentin, and cementum)	Complete demineralization (0.6 N HCl)	Parietal defect (ø 8 mm)/Approx. 13 weeks (90 days)	DDM: Significantly greater bone density than the ungrafted controls with low antigenicity for 13 weeks
2008 (Gomes et al.) [38]	Osteopromotion Optical density	48 New Zealand rabbits (Central incisor)	Slices, 8 mm in thickness (Consisting of enamel, dentin, and cementum)	Complete demineralization (0.6 HCl)	Parietal defect (ø 8 mm)/Approx. 13 weeks (90 days)	DDM: Dentin shows significantly greater radio-opacity and better trabecular bone arrangement than the empty negative controls during 13 weeks in a radiological study
2010 (Al-Namnam et al.) [44]	Osteocompatibility Quantitative comparison of bone formation	16 New Zealand white rabbits (Central incisor root)	Dentin particles 2–4 mm	Un-demineralization	Femoral defects (ø 5 mm)/12 weeks	MDM particle: No significant difference in new bone formation between autogenous bone graft, ungrafted sites, and MDM particles on histomorphometric analysis until 12 weeks.
2012 (Bormann et al.) [39]	Inflammatory and neovascularization response	24 isogenic mice (Mandibular central incisor)	Perforated (300 µm) dentin slices. 3 × 3 × 1 mm1 mm thick	Un-demineralization	Calvarial defect (36 mm^2^)/3 weeks (22 days)	Perforated MDM slice and ß-TCP scaffolds are similar to isogenic bone in terms of inflammatory and neovascularization response, highlighting their potential utility in the regeneration of bone defects.
2013 (Bakhshalian et al.) [40]	Osteopromotion	6 rabbits (Central incisor)	Dentin pieces 2 mm^3^	Complete demineralization (0.6 N HCl)	Parietal defect (ø 8 mm, 0.5 mm in depth)/15–90 days	The amount of bone regeneration: Significantly higher in the DDM group than in the ungrafted group.
2013 (Bakhshalian et al.) [41]	Osteopromotion Blood biomarkers	24 New Zealand white rabbits (Mandibular incisor)	Powders 300 µm in pooling	Complete demineralization (0.6 N HCl)	Skull defect (ø 8 mm, 0.5 mm in depth)/15–90 days	DDM: Significantly increased bone mass and improved bone quality without causing an inflammatory reaction or infection. WBC count: Higher in the early stage, but lower in the later stage than that in the empty control. ALP: There was no difference in the plasma.
2016 (Gomes et al.) [42]	Osteopromotion	60 adult New Zealand rabbits (Central incisor)	Slices, 8 mm in thickness (Consisting of enamel, dentin, and cementum)	Complete demineralization (0.6 N HCl)	Parietal defect (ø 8 mm)/Approx. 13 weeks (90 days)	ALP: Significantly higher in the DDM group than in the empty control, empty diabetic, and DDM–PRP groups, confirming the findings of intense osteoblastic activity and increased bone mineralization. DDM promoted superior bone architectural microstructure in bone defects in diabetic rabbits because of its effective osteoinductive and osteoconductive activity, whereas PRP stimulated angiogenesis and red bone marrow formation.
2018 (Um et al.) [43]	Osteopromotion	6 rabbits (Dentin)	Powder 300–800 µm	Partial demineralization (0.6 N HCl)	Calvarial defect (ø 8 mm)/1–4 weeks	DDM: Osteoinductive and osteoconductive function in a histological study.

Abbreviations: DDM—demineralized dentin matrix; MDM—mineralized dentin matrix; HCl—hydrochloric acid; ALP—alkaline phosphatase activity; PRP—platelet-rich plasma; WBC—white blood cells.

## Data Availability

Data sharing not applicable.

## References

[1] Avery S.J., Sadaghiani L., Sloan A.J., Waddington R.J. (2017). Analysing the bioactive makeup of demineralised dentine matrix on bone marrow mesenchymal stem cells for enhanced bone repair. Eur. Cell Mater..

[2] Urist M.R., Silverman B.F., Büring K., Dubuc F.L., Rosenberg J.M. (1967). The bone induction principle. Clin. Orthop. Relat. Res..

[3] Murata M., Okubo N., Shakya M., Kabir M., Yokozeki K., Zhu B., Ishikawa M., Kitamura R., Akazawa T. (2019). Dentin Materials as Biological Scaffolds for Tissue Engineering. Biomaterial-Supported Tissue Reconstruction or Regeneration.

[4] Kim Y.-K., Um I.-W., Murata M. (2014). Tooth Bank System for Bone Regeneration—Safety Report. J. Hard Tissue Biol..

[5] Murata M. (2005). Bone Engineering Using Human Demineralized Dentin Matrix and Recombinant Human BMP-2. J. Hard Tissue Biol..

[6] Kim Y.-K., Um I.-W., An H.-J., Kim K.-W., Hong K.-S., Murata M. (2014). Effects of Demineralized Dentin Matrix Used as an rhBMP-2 Carrier for Bone Regeneration. J. Hard Tissue Biol..

[7] Butler W.T., Mikulski A., Urist M.R., Bridges G., Uyeno S. (1977). Noncollagenous proteins of a rat dentin matrix possessing bone morphogenetic activity. J. Dent. Res..

[8] Kawai T., Urist M.R. (1989). Bovine tooth-derived bone morphogenetic protein. J. Dent. Res..

[9] Bessho K., Tagawa T., Murata M. (1990). Purification of rabbit bone morphogenetic protein derived from bone, dentin, and wound tissue after tooth extraction. J. Oral. Maxillofac. Surg..

[10] Bessho K., Tanaka N., Matsumoto J., Tagawa T., Murata M. (1991). Human dentin-matrix-derived bone morphogenetic protein. J. Dent. Res..

[11] Murata M. (2003). Autogenous demineralized dentin matrix for maxillary sinus augmentation in humans: The first clinical report. J. Dent. Res..

[12] Gomes M.F., Abreu P.P., Morosolli A.R., Araújo M.M., Goulart M. (2006). Densitometric analysis of the autogenous demineralized dentin matrix on the dental socket wound healing process in humans. Braz. Oral. Res..

[13] Kim Y.K., Kim S.G., Byeon J.H., Lee H.J., Um I.U., Lim S.C., Kim S.Y. (2010). Development of a novel bone grafting material using autogenous teeth. Oral. Surg. Oral. Med. Oral. Pathol. Oral. Radiol. Endod..

[14] Minetti E., Giacometti E., Gambardella U., Contessi M., Ballini A., Marenzi G., Celko M., Mastrangelo F. (2020). Alveolar Socket Preservation with Different Autologous Graft Materials: Preliminary Results of a Multicenter Pilot Study in Human. Materials.

[15] Li P., Zhu H., Huang D. (2018). Autogenous DDM versus Bio-Oss granules in GBR for immediate implantation in periodontal postextraction sites: A prospective clinical study. Clin. Implant. Dent. Relat. Res..

[16] Kim Y.K., Lee J.H., Um I.W., Cho W.J. (2016). Guided Bone Regeneration Using Demineralized Dentin Matrix: Long-Term Follow-Up. J. Oral. Maxillofac. Surg..

[17] Minetti E., Palermo A., Contessi M., Gambardella U., Schmitz J., Giacometti E., Celko M., Trisi P. (2019). Autologous tooth graft for maxillary sinus augmentation: A multicenter clinical study. Int. J. Growth Factors Stem. Cells Dent..

[18] Del Canto-Díaz A., de Elío-Oliveros J., Del Canto-Díaz M., Alobera-Gracia M.A., Del Canto-Pingarrón M., Martínez-González J.M. (2019). Use of autologous tooth-derived graft material in the post-extraction dental socket. Pilot study. Med. Oral. Patol. Oral. Y Cir. Bucal.

[19] Tanoue R., Ohta K., Miyazono Y., Iwanaga J., Koba A., Natori T., Iwamoto O., Nakamura K.I., Kusukawa J. (2018). Three-dimensional ultrastructural analysis of the interface between an implanted demineralised dentin matrix and the surrounding newly formed bone. Sci. Rep..

[20] Urist M.R. (1965). Bone: Formation by autoinduction. Science.

[21] Zhang M., Powers R.M., Wolfinbarger L. (1997). Effect(s) of the demineralization process on the osteoinductivity of demineralized bone matrix. J. Periodontol..

[22] Glowacki J. (2005). A review of osteoinductive testing methods and sterilization processes for demineralized bone. Cell Tissue Bank.

[23] Bang G., Urist M.R. (1967). Bone induction in excavation chambers in matrix of decalcified dentin. Arch. Surg..

[24] Yeomans J.D., Urist M.R. (1967). Bone induction by decalcified dentine implanted into oral, osseous and muscle tissues. Arch. Oral. Biol..

[25] Urist M.R., Dowell T.A., Hay P.H., Strates B.S. (1968). Inductive substrates for bone formation. Clin. Orthop. Relat. Res..

[26] Huggins C.B., Urist M.R. (1970). Dentin matrix transformation: Rapid induction of alkaline phosphatase and cartilage. Science.

[27] Huggins C., Wiseman S., Reddi A.H. (1970). Transformation of fibroblasts by allogeneic and xenogeneic transplants of demineralized tooth and bone. J. Exp. Med..

[28] Bang G. (1972). Induction of heterotopic bone formation by demineralized dentin in guinea pigs: Antigenicity of the dentin matrix. J. Oral. Pathol..

[29] Reddi A.H., Huggins C.B. (1973). Influence of geometry of transplanted tooth and bone on transformation of fibroblasts. Proc. Soc. Exp. Biol. Med..

[30] Bang G. (1973). Induction of heterotopic bone formation by demineralized dentin: An experimental model in guinea pigs. Scand. J. Dent. Res..

[31] Linden G.J. (1975). Bone induction in implants of decalcified bone and dentine. J. Anat..

[32] Nilsen R. (1977). Electron microscopy of induced heterotopic bone formation in guinea pigs. Arch. Oral. Biol..

[33] Inoue T., Deporter D.A., Melcher A.H. (1986). Induction of chondrogenesis in muscle, skin, bone marrow, and periodontal ligament by demineralized dentin and bone matrix in vivo and in vitro. J. Dent. Res..

[34] Pinholt E.M., Bang G., Haanaes H.R. (1990). Alveolar ridge augmentation by osteoinduction in rats. Scand. J. Dent. Res..

[35] Bang G., Nordenram Å., Anneroth G. (1972). Allogenic demineralized dentin implants in jaw defects of Java monkeys. Int. J. Oral. Surg..

[36] Carvalho V.A., Tosello Dde O., Salgado M.A., Gomes M.F. (2004). Histomorphometric analysis of homogenous demineralized dentin matrix as osteopromotive material in rabbit mandibles. Int. J. Oral. Maxillofac. Implant..

[37] Gomes M.F., Banzi E.C., Destro M.F., Lavinicki V., Goulart M. (2007). Homogenous demineralized dentin matrix for application in cranioplasty of rabbits with alloxan-induced diabetes: Histomorphometric analysis. Int. J. Oral. Maxillofac. Implant..

[38] Gomes M.F., Destro M.F., Banzi E.C., Vieira E.M., Morosolli A.R., Goulart M. (2008). Optical density of bone repair after implantation of homogenous demineralized dentin matrix in diabetic rabbits. Braz. Oral. Res..

[39] Bormann K.H., Suarez-Cunqueiro M.M., Sinikovic B., Kampmann A., von See C., Tavassol F., Binger T., Winkler M., Gellrich N.C., Rücker M. (2012). Dentin as a suitable bone substitute comparable to ß-TCP—An experimental study in mice. Microvasc. Res..

[40] Bakhshalian N., Hooshmand S., Campbell S.C., Kim J.S., Brummel-Smith K., Arjmandi B.H. (2013). Biocompatibility and microstructural analysis of osteopromotive property of allogenic demineralized dentin matrix. Int. J. Oral. Maxillofac. Implant..

[41] Bakhshalian N., Jalayer T., Shahoon H., Arjmandi B.H., Azimi H.R. (2013). Osteopromotive property of allogenic demineralized dentin matrix: A pilot study. J. West. Soc. Periodontol. Periodontal Abstr..

[42] Gomes M.F., Valva V.N., Vieira E.M., Giannasi L.C., Salgado M.A., Vilela-Goulart M.G. (2016). Homogenous demineralized dentin matrix and platelet-rich plasma for bone tissue engineering in cranioplasty of diabetic rabbits: Biochemical, radiographic, and histological analysis. Int. J. Oral. Maxillofac. Surg..

[43] Um I.-W., Kim Y.-K., Jun S.-H., Kim M.-Y., Cui N. (2018). Demineralized Dentin Matrix as a Carrier of Recombinant Human Bone Morphogenetic Proteins: In vivo Study. J. Hard Tissue Biol..

[44] Al-Namnam N., Shanmuhasuntharam P., Ha K.O., Siar C.H. (2010). Processed allogenic dentine as a scaffold for bone healing: An in vivo study. Aust. J. Basic Appl. Sci..

[45] Um I.W., Ku J.K., Kim Y.K., Lee B.K., Leem D.H. (2020). Histological Review of Demineralized Dentin Matrix as a Carrier of rhBMP-2. Tissue Eng. Part. B Rev..

[46] Dubuc F.L., Urist M.R. (1967). The accessibility of the bone induction principle in surface-decalcified bone implants. Clin. Orthop. Relat. Res..

[47] Urist M.R., Iwata H., Strates B.S. (1972). Bone morphogenetic protein and proteinase in the guinea pig. Clin. Orthop. Relat. Res..

[48] Masaru M. (2012). Collagen biology for bone regenerative surgery. J. Korean Assoc. Oral. Maxillofac. Surg..

[49] Fuentes G.C., Newgren J. (2008). Physiology and clinical pathology of laboratory new zealand white rabbits housed individually and in groups. J. Am. Assoc. Lab. Anim. Sci..

[50] Urist M.R., Mikulski A., Boyd S.D. (1975). A chemosterilized antigen-extracted autodigested alloimplant for bone banks. Arch. Surg..

[51] Horowitz M.C., Friedlaender G.E. (1991). Induction of specific T-cell responsiveness to allogeneic bone. J. Bone Jt. Surg. Am..

[52] Mikulski A.J., Urist M.R. (1975). An antigenic antimorphogenetic bone hydrophobic glycopeptide (AHG). Prep. Biochem..

[53] Russell J.L., Block J.E. (1999). Clinical utility of demineralized bone matrix for osseous defects, arthrodesis, and reconstruction: Impact of processing techniques and study methodology. Orthopedics.

[54] Um I.W., Ku J.K., Lee B.K., Yun P.Y., Lee J.K., Nam J.H. (2019). Postulated release profile of recombinant human bone morphogenetic protein-2 (rhBMP-2) from demineralized dentin matrix. J. Korean Assoc. Oral Maxillofac. Surg..

[55] Murata M., Akazawa T., Mitsugi M., Um I.W., Kim K.W., Kim Y.K. (2011). Human dentin as novel biomaterial for bone regeneration. Pignatello R.

[56] Ike M., Urist M.R. (1998). Recycled dentin root matrix for a carrier of recombinant human bone morphogenetic protein. J. Oral. Implant..

[57] Koga T., Minamizato T., Kawai Y., Miura K.-I., I T., Nakatani Y., Sumita Y., Asahina I. (2016). Bone Regeneration Using Dentin Matrix Depends on the Degree of Demineralization and Particle Size. PLoS ONE.

[58] Um I.W., Kim Y.K., Mitsugi M. (2017). Demineralized dentin matrix scaffolds for alveolar bone engineering. J. Indian Prosthodont. Soc..

[59] Rijal G., Shin H.I. (2017). Human tooth-derived biomaterial as a graft substitute for hard tissue regeneration. Regen. Med..

[60] Friedenstein A.J., Chailakhyan R.K., Gerasimov U.V. (1987). Bone marrow osteogenic stem cells: In vitro cultivation and transplantation in diffusion chambers. Cell Tissue Kinet..

[61] Owen M. (1988). Marrow stromal stem cells. J. Cell Sci. Suppl..

[62] Folkman J., Greenspan H.P. (1975). Influence of geometry on control of cell growth. Biochim. Et Biophys. Acta (BBA) Rev. Cancer.

[63] Kim Y.K., Pang K.M., Yun P.Y., Leem D.H., Um I.W. (2017). Long-term follow-up of autogenous tooth bone graft blocks with dental implants. Clin. Case Rep..

[64] Moon Y.S., Sohn D.S., Kim G., Park I. (2019). Comparative Histomorphometric Evaluation of Bone Regeneration with Different Preparations of Xenogeneic Tooth Block Bone. Int. J. Oral. Maxillofac. Implant..

[65] Kabir M.A., Murata M., Akazawa T., Kusano K., Yamada K., Ito M. (2017). Evaluation of perforated demineralized dentin scaffold on bone regeneration in critical-size sheep iliac defects. Clin. Oral. Implant. Res..

[66] Glowacki J., Altobelli D., Mulliken J.B. (1981). Fate of mineralized and demineralized osseous implants in cranial defects. Calcif. Tissue Int..

[67] Dozza B., Lesci I.G., Duchi S., Della Bella E., Martini L., Salamanna F., Falconi M., Cinotti S., Fini M., Lucarelli E. (2017). When size matters: Differences in demineralized bone matrix particles affect collagen structure, mesenchymal stem cell behavior, and osteogenic potential. J. Biomed. Mater. Res. A.

[68] Nam J.W., Kim M.Y., Han S.J. (2016). Cranial bone regeneration according to different particle sizes and densities of demineralized dentin matrix in the rabbit model. Maxillofac. Plast Reconstr. Surg..

[69] Li R., Guo W., Yang B., Guo L., Sheng L., Chen G., Li Y., Zou Q., Xie D., An X. (2011). Human treated dentin matrix as a natural scaffold for complete human dentin tissue regeneration. Biomaterials.

[70] Kim Y.K., Kim S.G., Yun P.Y., Yeo I.S., Jin S.C., Oh J.S., Kim H.J., Yu S.K., Lee S.Y., Kim J.S. (2014). Autogenous teeth used for bone grafting: A comparison with traditional grafting materials. Oral. Surg. Oral. Med. Oral. Pathol. Oral. Radiol..

